# Path Analysis to Identify Factors Influencing Health Skills and Behaviors in Adolescents: A Cross-Sectional Survey

**DOI:** 10.1371/journal.pone.0104406

**Published:** 2014-08-08

**Authors:** Xiaohua Ye, Zhenjiang Yao, Weidong Liu, Yanping Fan, Ya Xu, Sidong Chen

**Affiliations:** 1 Guangdong Key Laboratory of Molecular Epidemiology, School of Public Health, Guangdong Pharmaceutical University, Guangzhou, Guangdong, China; 2 School of Public Health and Tropical Medicine, Southern Medical University, Guangzhou, Guangdong, China; Leibniz Institute for Prevention Research and Epidemiology (BIPS), Germany

## Abstract

**Background:**

Studies conducted in the past mostly rely on models of functional health literacy in adult populations. However, such models do not satisfy the need for health intervention in adolescents. The identification of key factors influencing adolescents' health literacy is essential in developing effective prevention and intervention measures. This study aimed to test a theoretical model of predictors on health skills and health behaviors in adolescents.

**Methods:**

A cross-sectional survey was conducted in Guangdong using a multi-stage stratified cluster sample design. A representative random sample of 3821 students aged 13–25 years was selected using multi-stage stratified cluster sampling. The path analysis was used to test a hypothesized model of health literacy.

**Results:**

The path analysis showed that knowledge of infectious disease (β = 0.26), health skills (β = 0.22), health concept (β = 0.20), general health knowledge (β = 0.15), gender (β = 0.12), and school performance (β = 0.06) had positive direct effect on health behaviors in adolescents. The explanatory variables accounted for 43% of the variance in explaining health behaviors. Knowledge of infectious disease (β = 0.30), health concept (β = 0.17), general health knowledge (β = 0.13), and school performance (β = 0.05) had positive indirect effect on health behaviors through the impacts on health skills.

**Conclusion:**

This study identified several direct and indirect factors influencing health skills and health behaviors in adolescents. These findings will assist health professionals designing effective health interventions that aim to improve health skills and health behaviors in adolescents.

## Introduction

Health literacy has become an important public health issue and also an important public health goal. While there is a large body of literature of health literacy on adults [1]–[4], few studies were focused on adolescents. Health literacy focusing on adolescents is necessary because of two main reasons. 1), studies have shown that many adolescents are reading below grade level. While adolescents are interested in understanding health information, they may find it difficult [5]–[9]. Adolescents with limited education may not be able to fully benefit from educational programs or health interventions due to their inability to understand or access the messages [10], [11]. In addition, adolescents with low literacy level were more likely to use weapons and less likely to seek health care for sexually transmitted diseases [12], [13]. This is a serious problem for adolescents. 2), adolescents are at a crucial stage of development characterized by many physical and psychosocial changes. Adolescents' experience advances in capacity for processing information, thinking more about abstract ideas and using reasoning skills. These capacities will carry with them into adulthood. To better understand the extent that adolescents understand health literacy, a cross-sectional survey among in-school adolescents was carried out from September 2009 to November 2010. The path analysis was conducted to explore several direct and indirect factors that potentially influence the health skills and health behaviors.

## Methods

### Ethics statement

This study was approved by the ethics committee of Guangdong Pharmaceutical University. The survey was qualified as involving no risks to participants. The goals of the study were given to the participants and they should express their willingness to participate. Written informed consents were obtained from parents or guardians for participants under age 18, or from participants who are over 18 years old. All procedures were approved by the ethics committee of Guangdong Pharmaceutical University.

### Study design and sampling

The target population was in-school adolescents aged 13–25 years old. A multi-stage, stratified cluster sampling of in-school students was conducted in Guangdong province, China. First, eight cities were randomly sampled from 21 cities in Guangdong province. Second, one high school was randomly drawn from each sampled city. As most universities were in the capital city (Guangzhou city), we randomly sampled 7 universities in Guangzhou. Finally, we selected a specific number of classes to reach a respondent sample size of 200 in each high school and 300 in each university.

### Measurement of health literacy

The questionnaire of "2009 health literacy survey of Chinese citizens" (Appendix S1) [14] prepared by the Ministry of Health of the People's Republic of China was used for the survey. The questionnaire covered six broad categories: (1) socio-demographic variables; (2) general knowledge; (3) knowledge on infectious diseases; (4) health concept; (5) health skills; and (6) health behaviors. The socio-demographic variables were age (years), gender, school performance (poor, good), student classification (high school students, university students), prestigious school (yes, no), region (rural, urban), only-child (yes, no) and monthly pocket money (<50 RMB, 50–99 RMB, 100–199 RMB, ≥200 RMB). For single-answer questions of health literacy, a correct response was scored as one point, and incorrect, “do not know” or missing responses were scored with zero point. For multiple-answer questions, the correct response rate equal to or greater than 60% was scored as one point, and else were scored with zero point. We measured the general knowledge with six questions (including early signals of cancer, influenza prevention, the normal value scope of blood pressure/axillary temperature/pulse frequency, and the law on prevention and control of occupational disease), and the total score ranged from 0 to 6. Similarly, we measured knowledge on infectious diseases, health concept, health skills and health behaviors with twelve, nine, ten, and fourteen questions respectively. The knowledge on infectious diseases included the routes of HIV transmission, children vaccination, treatment for tuberculosis symptoms and vector-borne diseases. Health concept included the concept of health, healthy lifestyle, and realization of health hazards associated with passive and active smoking. Health skills referred to individual skills in dealing with unexpected events and diseases, including knowing the emergency medical call, having the ability to identify the flammable/high pressure/radioactive/biosecurity/explosive/toxic logos and OTC marked on medicine boxes, and having an ability to deal with fires and measure body temperature. Health behaviors asked about individual behaviors and actions including habits of using towels and brushing teeth, physical examination, alcohol intake, salt intake, antibiotics use, use of cutting board, rabies vaccine, safe driving, infant feeding and so on. The total scores for each type of health literacy were created separately by summing up the scores of questions. Higher scores indicated greater degree of health literacy.

### Data collection and quality control

All interviewers in each area were trained to ensure that the survey was carried out according to the protocol and that operation procedures were identical across all areas. After obtaining informed consent, eligible students were asked to complete a face-to-face survey by trained interviewers. Participants were assured of anonymity by coding each questionnaire without using names or personal identifiers. In order to evaluate the feasibility of investigation, a pilot study was carried out before formal investigation. All data were entered into computer by trained data-entry personnel. Data quality was assured by using double data entry procedures and a system to automatically detect data entry errors. Before data analysis, data cleaning was carried out to ensure data quality.

### Hypothesis of a health literacy model

Previous studies [3], [6], [10], [15]–[21] have given the relationship between socio-demographic characteristics, health knowledge, health skills, health behaviors and health outcomes, they are all theoretical explanations. Few studies have tried to validate them through the use of statistical modeling. So this study aimed to develop a health literacy model and to statistically validate it using path analysis. With the models of previous studies for reference, we proposed a health literacy model. In this model, socio-demographic indicators, including age, gender, school performance, student classification, prestigious school, region, only-child and monthly pocket money, are the basic factors influencing other variables. Besides socio-demographic indicators, health knowledge (including general knowledge and infectious knowledge) and health concept also influence the development of health skills. Then health skills have direct effect on health behaviors, meanwhile, as a mediator among health knowledge, health concept and health behaviors.

### Data analysis

The Cronbach's α (good at coefficient≥0.7) was calculated to examine internal consistency of the questionnaire and construct validity was assessed through Pearson's correlations (good at coefficient≥0.7) [22]–[24]. Path analysis was used to identify factors influencing health skills and behaviors in adolescents [25]–[27]. Firstly, we checked variables to ensure they met assumptions of normal distribution and multicollinearity [28]. If multicollinearity diagnostics (variance inflation factor, VIF) were less than 5 or bivariate correlations did not exceed 0.80, the assumption of reasonable independence among predictor variables was met. Secondly, the path model was used to test the direct and indirect relations among the variables. Health behavior was the dependent variable. Exogenous independent variables were general knowledge, knowledge on infectious diseases, health concept and socio-demographic variables. Endogenous independent variable was health skills. Goodness of fit of the final model was assessed by chi-square test and the goodness of fit indices, such as Root Mean Square Error of Approximation (RMSEA), goodness-of-fit index (GFI), adjusted goodness-of-fit index (AGFI), normed fit index (NFI), relative fit index (RFI), incremental fit index (IFI), Tacker-Lewis index (TLI) and comparative fit index (CFI). Values for GFI, AGFI, NFI, RFI, IFI, TLI and CFI range from 0 to 1 with recommending values greater than 0.90 indicating a good fit. Conventionally, there is a good fit if RMSEA is less than 0.05, and there is adequate fit if RMSEA is less than 0.08 [14], [26]. Descriptive analyses were conducted using SPSS/WIN 13.0 (SPSS, Inc.) and the path models were analyzed using SPSS AMOS18.0 (SPSS, Inc.).

## Results

### Enrollment and characteristics of students

A total of 3821 students were interviewed, and the effective response rate was 98.2% (3754/3821). Participants' age ranged from 13 to 25 (mean = 18.9, standard deviance [SD] = 2.5) years old. Table 1 showed the characteristics of the participated students. More than half of the students were males. One third of the students were from high schools and two-third of them from universities. Over 90% of the students reported good school performance. Half of the students were from prestigious schools.

**Table 1:** Baseline characteristics.

Baseline variables	Category or range	n	Per cent
Gender	Female	1577	42.0
	Male	2177	58.0
School performance	Poor	340	9.1
	Good	3414	90.9
Student classification	High school students	1606	42.8
	University students	2148	57.2
Prestigious school	No	1906	50.8
	Yes	1848	49.2

n, number of participants surveyed.

### The description of health literacy

The internal consistency of health literacy was adequate (α = 0.86). There were statistically significant correlations of the total scores of health literacy (the sum of five subtypes in health literacy questionnaire) with the score of general knowledge (r = 0.66, p = 0.00), knowledge of infectious disease (r = 0.80, p = 0.00), health concept (r = 0.70, p = 0.00), health skills (r = 0.72, p = 0.00) and health behaviors (r = 0.81, p = 0.00), supporting the validity of the scales. The mean scores (±SD) for the general knowledge, knowledge of infectious disease, health concept, health skills and health behaviors were 4.4±1.4, 8.1+2.3, 6.7±1.7, 7.8±2.2 and 8.5±2.4 respectively (Table 2).

**Table 2:** Description statistics of health literacy (n = 3754).

Variables	Range	Mean	SD
General knowledge	0–6	4.1	1.4
Knowledge on infectious diseases	0–12	8.1	2.3
Health concept	0–9	6.7	1.7
Health skills	0–10	7.8	2.2
Health behaviors	0–14	8.5	2.4

SD, standard deviation.

### Path analysis

Multicollinearity was not detected as bivariate correlations did not exceed 0.80 and each VIF was less than 5. The skews of all variables were less than 3, and the coefficients of kurtosis of all variables were less than 8. Thus, no violation of assumption of normality was detected.

**Figure 1:**
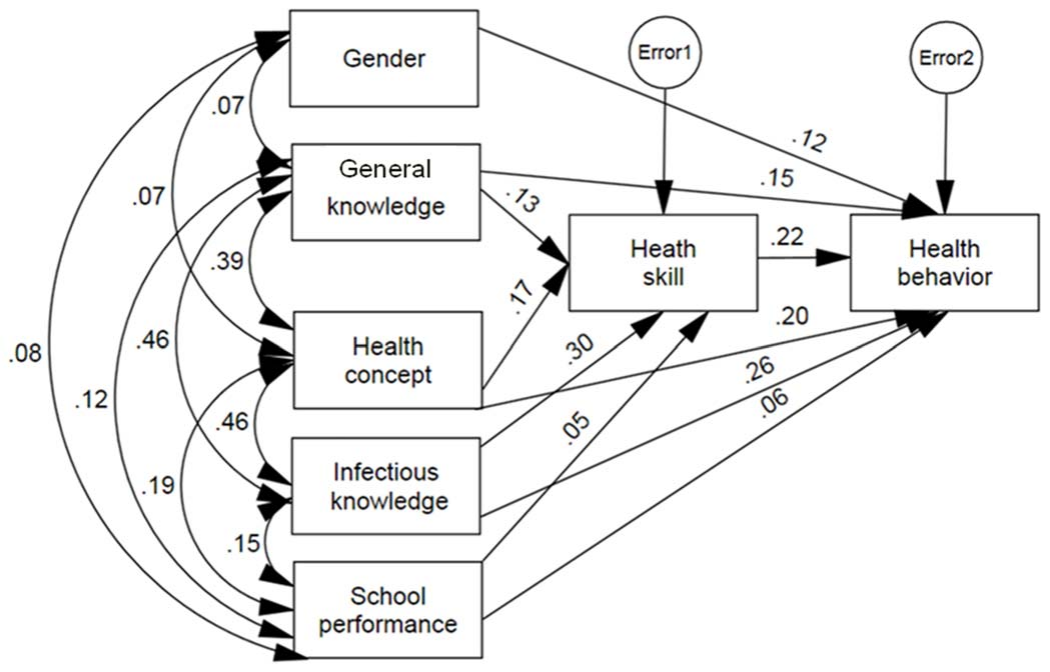
Final Path Model (standardized regression coefficients and correlation between variables).

Path coefficients were calculated by a series of multiple regression analyses based on the hypothesized model. The final results were presented in Table 3 and Figure 1. The final model had a good fit with chi-square  = 2.2 (df = 2, P = 0.325), GFI = 1.0, AGFI = 0.998, NFI = 1.0, RFI = 0.996, IFI = 1.0, TLI = 1.0, CFI = 1.0 and RMSEA = 0.006. The knowledge of infectious disease (β = 0.26), health skills (β = 0.22), health concept (β = 0.20) and general knowledge (β = 0.15) had positive direct effect on health behaviors. The results also showed that female gender (β = 0.12) and good school performance (β = 0.06) had positive direct effect on health behaviors. The explanatory variables accounted for 43% of the variance in explaining health behaviors. In addition, knowledge of infectious disease (β = 0.30), health concept (β = 0.17), basic knowledge (β = 0.13) and school performance (β = 0.05) had positive indirect effect on health behaviors through their effect on health skills. The model variables accounted for 24% of the variance of health skills.

**Table 3:** Path coefficients based on the final model.

Outcome variables	R^2^	Predictor variables	Unstandardized coefficient estimate	SE	p-value	Standardized coefficient estimate
Health behaviors	0.43	Health skills	0.246	0.016	<0.001	0.222
		Health concept	0.277	0.021	<0.001	0.195
		Knowledge on infectious diseases	0.266	0.016	<0.001	0.259
		General knowledge	0.265	0.025	<0.001	0.153
		School performance	0.396	0.079	<0.001	0.064
		gender	0.574	0.060	<0.001	0.119
Health skills	0.24	Health concept	0.215	0.021	<0.001	0.169
		Knowledge on infectious diseases	0.279	0.016	<0.001	0.301
		General knowledge	0.196	0.026	<0.001	0.126
		School performance	0.267	0.081	0.001	0.047

SE, standard error.

## Discussion

This study found that general knowledge, knowledge of infectious disease, health concept and school performance had direct and indirect effects on health behaviors and health skills. These results could assist health professionals for designing effective health interventions to improve adolescents' health skills and health behaviors, especially for students of male and poor school performance.

### Direct effects

The results of path model revealed that health skills, health concept, general knowledge and knowledge of infectious disease had direct effects on health behaviors, which were consistent with findings from previous studies [3], . One study from Japan indicated that individuals with less health skills had a significantly higher rate of smoking, and were more likely to have a history of hypertension [3]. Taiwanese adolescents with low health literacy were also found to be less likely to exhibit health-promoting behaviors (AOR = 0.58, 95%CI = 0.39–0.86) [15]. Findings from two longitudinal studies suggested that early hyperactivity was associated with school difficulties, attention problems and poor reading in adolescence [6]. It was also important to note that knowledge of infectious disease had the largest direct effect on health behaviors.

The results indicated that socio-demographic (such as gender and school performance) had direct effect on health behaviors. Consistent with other studies [18], [19], female gender was found to relate with higher health behaviors, which indicated lower health behavior level for male students. Female participants were also associated with better general knowledge and health concept, which was also consistent with previous studies [10], [19], [20]. In this study, those with good school performance had significant higher level of health behaviors, general knowledge, knowledge of infectious disease, health concept and health skills, which was similar with the previous study [21]. Possible explanations for above associations included that male and poor school performance students had poor self-discipline, concern less about daily health behaviors, and were unwilling to disclose and communicate the health problems with classmates, friends and parents.

It was noted that the path model accounted for 43% of the variance in health behaviors, indicating that other factors should be included to more fully explain the outcome. Future model construction should combine other variables to assess, for example, cultural background [29], peer and parental influence [30], [31] and media use [32], [33] et al.

### Indirect effects

This path model indicated that general knowledge, knowledge of infectious disease and health concept had positive indirect effect on health behaviors through their impacts on health skills. To note, the association of health skills with knowledge of infectious disease was much greater than that with general knowledge and health concept. Consistent with this study, one research demonstrated that women with lower health literacy aged 16–21 years had lower comprehension of health information [34]. Apart from the direct effect on health behaviors, school performance also had positive indirect effect on health behaviors through their impacts on health skills.

### Strengths and Limitations

Path analysis is superior to linear regression analysis as it provides an explanation of the relation and the relative importance of each factor, and examines the direct and indirect relations among the variables. The most meaningful advantage of this study was that it contributed additionally to the literature by exploring several direct and indirect influencing factors of health skills and health behaviors in adolescents. Some potential limitations also should be considered in interpreting the findings. Firstly, the study design was cross-sectional, so we can only describe associations among the factors studied, not causal relations. Prospective studies are needed to show the causality. Secondly, this was a school-based study and non-student youth were not included. Thus its generalization was limited. Nonetheless, this study was among the first to examine pathways used to influence health behaviors and health skills in a homogenous, large study of in-school adolescents.

In conclusion, this study found that general knowledge, knowledge of infectious disease, health concept and school performance had direct and indirect effect on health behaviors and health skills in adolescents. This study warrants further investigation on health literacy in adolescents, perhaps with the use of a prospective design. The results of this study are especially useful when developing effective interventions addressing health literacy in adolescents.

## Supporting Information

Appendix S1 **Health Literacy Survey Questions.**
(DOC)
