# Supplementary material for: Path Analysis to Identify Factors Influencing Health Skills and Behaviors in Adolescents: A Cross-Sectional Survey
Source: PLoS One. 2014 Aug 8;9(8):e104406. doi: 10.1371/journal.pone.0104406 (PMC4126710; doi:10.1371/journal.pone.0104406)
Supplement: Appendix S1 — Health Literacy Survey Questions. (DOC) [file pone.0104406.s001.doc]

**Appendix S1**. **Health Literacy Survey Questions**

**1. General knowledge:**

Do you think weight loss is an early signal of cancer?

① Yes ② No ③ Do not know

Do you think hand-washing can help you prevent influenza?

① Yes ② No ③ Do not know

Which option belong to the normal value scope of blood pressure（high / low pressure）?

① 140/95 mmHg ② 120/80 mmHg ③ 150/100 mmHg ④ Do not know

Which option is the normal value scope of axillary temperature in adults?

① 35-36℃ ② 36-37℃ ③ 37-38℃ ④ Do not know

Which option is the normal value scope of pulse frequency in adults?

① 30-50 beats/min ② 60-100 beats/min ③ 100-120 beats/min ④ Do not know

Is there a law dedicating to the protection of the right of workers engaged in toxic work?

① Yes ② No ③ Do not know

**2. Knowledge of infectious diseases:**

Can HIV be transmitted through blood and blood products？

① Yes ② No ③ Do not know

Can HIV be transmitted through mosquito bites？

① Yes ② No ③ Do not know

Can HIV be transmitted through sharing needles？

① Yes ② No ③ Do not know

Can HIV be transmitted through breastfeeding？

① Yes ② No ③ Do not know

Can use of condoms reduce the risk of HIV infection?

① Yes ② No ③ Do not know

Which kind of diseases can be prevented by children vaccination?

① Infectious diseases ② Chronic diseases ③ Malnutrition ④ Do not know

When you have the symptoms of expectoration for more than two weeks, hemoptysis, low fever, fatigue and night sweats, the best solution is:

① See a doctor only when feeling serious ② Buy yourself some cold medicine

③ See a doctor as soon as possible ④ Do not know

Do you think the treatment for tuberculosis is free?

① Yes ② No ③ Not necessarily ④ Do not know

Which is a mosquito-borne disease?

① Malaria ② Plague ③ Dysentery ④ Do not know

Which is a fly-borne disease?

① Malaria ② Plague ③ Dysentery ④ Do not know

Which is a rodent-borne disease?

① Malaria ② Plague ③ Dysentery ④ Do not know

Which is a cockroach-borne disease?

① Malaria ② Plague ③ Dysentery ④ Do not know

**3. Health concept:**

Do you agree that heath refers to physically strong and no physical illnesses?

① Yes ② No ③ Do not know

Do you agree that people with good health needn’t pay attention to health issues?

① Yes ② No ③ Do not know

Do you agree that the more the body fat is, the better body becomes?

① Yes ② No ③ Do not know

Do you think the therapeutic effect of injection is better and faster than that of medication?

① Yes ② No ③ Do not know

Do you think it is harmless for adults to donor 200ml blood?

① Yes ② No ③ Do not know

Do you think psychological problems are not illnesses?

① Yes ② No ③ Do not know

The healthy lifestyle should include:

① Quit smoking and limit alcohol intake ② Balanced diet ③ Live in harmony with others

④ Eat more health food ⑤ Control the weight ⑥ Eat and sleep more

⑦ Regular exercise ⑧ Do not know

Which kinds of diseases are caused by passive smoking?

① Lung cancer ② Coronary heart disease ③ Chronic obstructive pulmonary disease

④ Tooth decay ⑤ Cataract ⑥ Affecting fetal development ⑦ Do not know

Which are the correct statements of quit smoking?

① The sooner, the better  ② Be good for health ③ Unable to successfully quit smoking

④ Older people needn’t quit smoking ⑤ Do not know

**4. Health skills:**

Which is the emergency medical call?

① 120 ② 119 ③ 122 ④ Do not know

Do you know the meaning of OTC marked on the right corner of medicine boxes?

① Drugs that must be prescribed by a doctor

② Drugs that can be bought without a doctor’s prescription

③ Do not know

Which is the flammable logo?

| 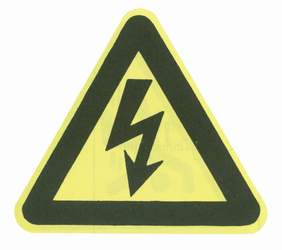① | 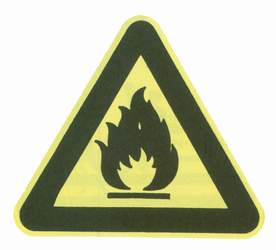② | 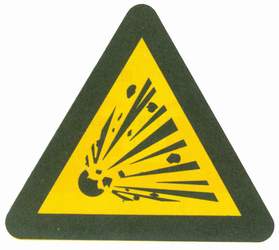③ | 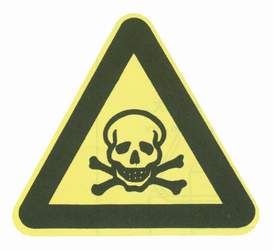④ | 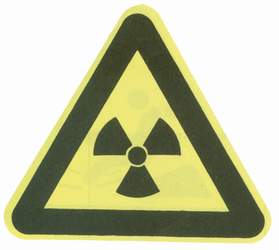⑤ | 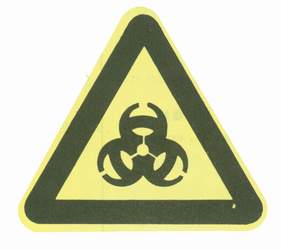⑥ |
| --- | --- | --- | --- | --- | --- |

Which is the high pressure logo?

| 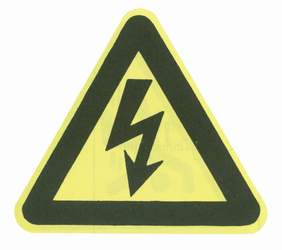① | 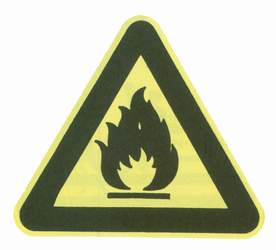② | 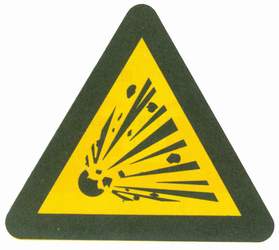③ | 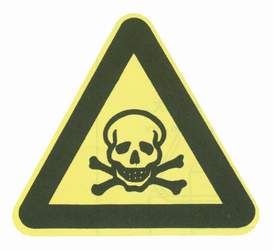④ | 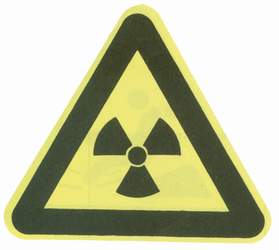⑤ | 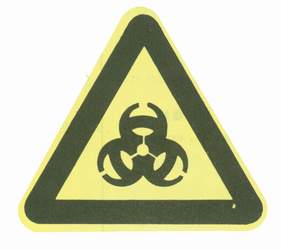⑥ |
| --- | --- | --- | --- | --- | --- |

Which is the radioactive logo?

| 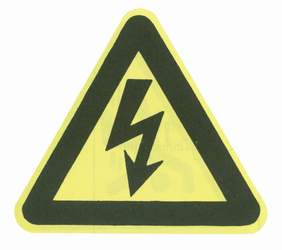① | 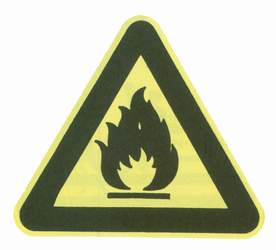② | 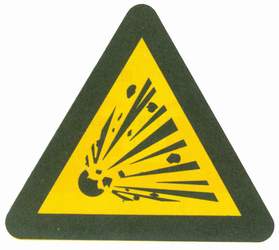③ | 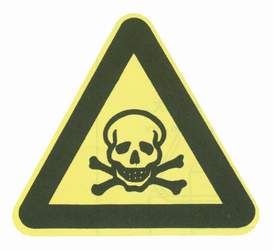④ | 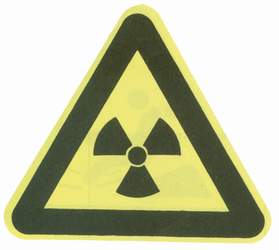⑤ | 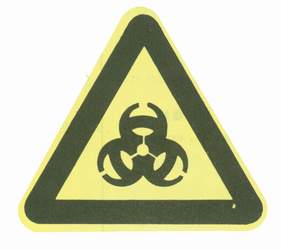⑥ |
| --- | --- | --- | --- | --- | --- |

Which is the biosecurity logo?

| 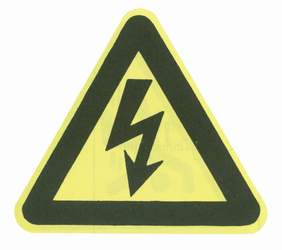① | 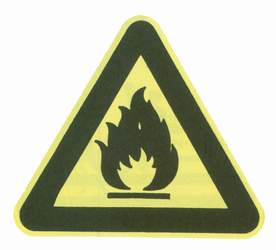② | 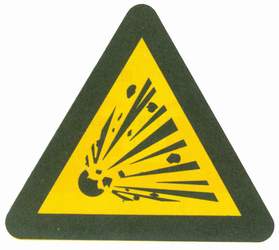③ | 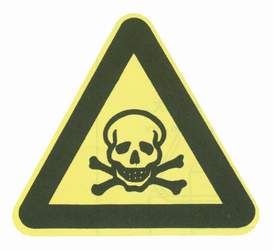④ | 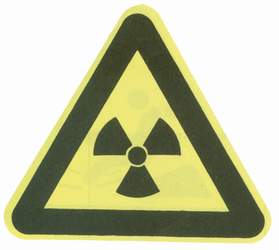⑤ | 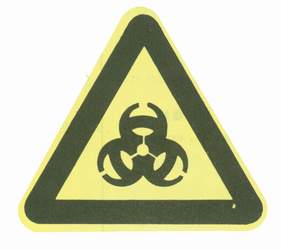⑥ |
| --- | --- | --- | --- | --- | --- |

Which is the explosive logo?

| 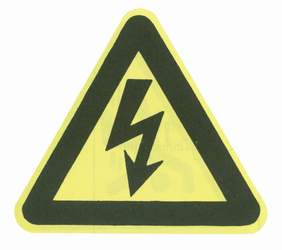① | 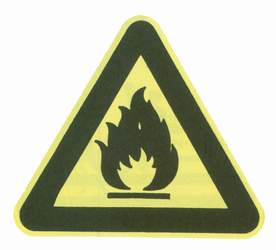② | 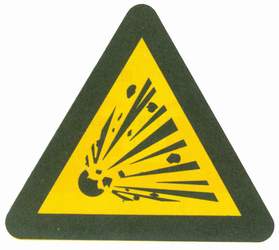③ | 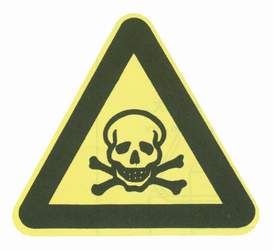④ | 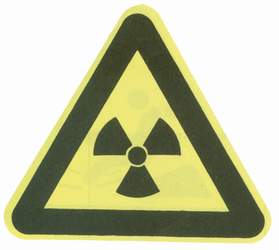⑤ | 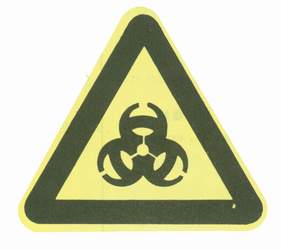⑥ |
| --- | --- | --- | --- | --- | --- |

Which is the toxic logo?

| 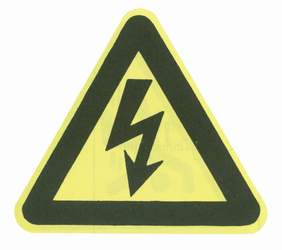① | 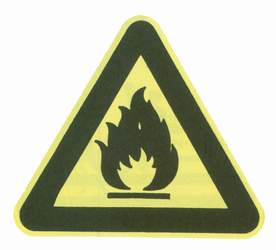② | 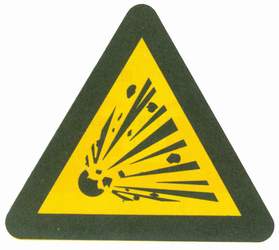③ | 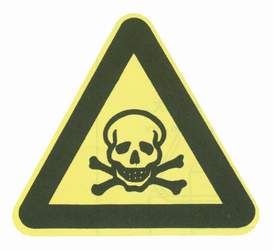④ | 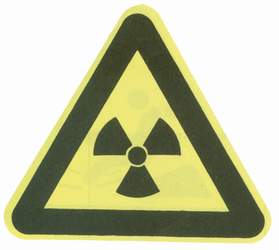⑤ | 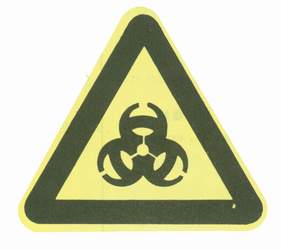⑥ |
| --- | --- | --- | --- | --- | --- |

What should we do if there was a fire?

① Cover your mouth and nose with a wet towel ② Fell down and evacuate from fire

③ Immediately call 119 ④ Take the elevator to escape as soon as possible

⑤ Carry valuables and escape as soon as possible ⑥ Do not know

Please demonstrate how to correctly measure body temperature.

1. Do it correctly
2. Do it incorrectly

③ Do not know

**5. Health behaviors:**

Which is your habit of using towels?

① Share towels with others ② Not share towels with others

Do you have at least once physical examination every year?

① Yes ② No

Which is your habit of brushing teeth?

① Brushing teeth only in the morning ② Brushing teeth only at night

③ Brushing teeth both in the morning and at night ④ Less than once a day

Which are the correct statements of antibiotics use?

1. Take antibiotics immediately when catching a cold

② Buy antibiotics without a doctor’s prescription

③ Antibiotics should be used under the guidance of a doctor

④ Antibiotics can kill bacteria and viruses ⑤ Do not know

Which is a correct statement on the use of cutting board?

① Use the same cutting board to cut raw and cooked food

② Use different cutting boards to cut raw and cooked food

③ Do not know

Which is a correct statement on expired food?

① Expired food can still eat as long as it is not bad

② Do not eat expired food

③ After being heated, the expired food is still safe to eat

④ Do not know

The physical examination frequency of women during pregnancy is:

① At least 3 times ② At least 5 times ③ At least 7 times

④ At least 9 times ⑤ Do not know

When bitten by cats or dogs, you should:

① Inject rabies vaccine immediately

② Wash the wound immediately with soap and water, and inject rabies vaccine as soon as possible

③ Wash the wound with water

④ Be treated by folk recipes

⑤ Do not know

Which is a correct statement on safe driving modes?

① Drive when only drink a little wine ② Must wear a motorcycle helmet

③ Drive without a seatbelt ④ Drive on speeding when there is little traffic on this road

⑤ Do not know

Which are the correct statements on infant feeding?

① Formula milk is more nutritious than breast milk

② The sooner, the better

③ Complementary feeding begins when the child was born six months

④ Complementary feeding should first add the cereal

⑤ Do not know

Which are the correct statements on dealing with gas poisoning?

① Open the window

② Move the people to air fresh and well ventilated place as soon as possible

③ Call an ambulance telephone

④ Move the people to cool place to cool down and pour vinegar

⑤ Do not know

Which is the healthy dose of alcohol intake in male adults？

① ≤25g ② ≤50g ③≤100g ④ ≤200g ⑤ Do not know

Which is the healthy dose of salt intake?

① ≤2g ② ≤6g ③ ≤9g ④ ≤12g ⑤ Do not know

Which are the diseases caused by contaminated water?

① Hepatitis A ② Hepatitis B ③ Poisoning ④ Cancer

⑤ Dysentery ⑥ Measles ⑦ Do not know
